# 2-O, 3-O desulfated heparin (ODSH) increases bacterial clearance and attenuates lung injury in cystic fibrosis by restoring HMGB1-compromised macrophage function

**DOI:** 10.1186/s10020-021-00334-y

**Published:** 2021-07-16

**Authors:** Mao Wang, Alex G. Gauthier, Thomas P. Kennedy, Haichao Wang, Uday Kiran Velagapudi, Tanaji T. Talele, Mosi Lin, Jiaqi Wu, LeeAnne Daley, Xiaojing Yang, Vivek Patel, Sung Soo Mun, Charles R. Ashby, Lin L. Mantell

**Affiliations:** 1 Department of Pharmaceutical Sciences, St. John’s University College of Pharmacy and Health Sciences, Queens, 11439 NY USA; 2 Wake Forest University School of Medicine, Winston Salem, NC USA; 3 The Feinstein Institute for Medical Research, Northwell Health System, Manhasset, NY USA

**Keywords:** Cystic fibrosis, Pulmonary infection, HMGB1, Macrophage functions, Phagocytosis, ODSH, NO

## Abstract

**Background:**

High mobility group box 1 protein (HMGB1) is an alarmin following its release by immune cells upon cellular activation or stress. High levels of extracellular HMGB1 play a critical role in impairing the clearance of invading pulmonary pathogens and dying neutrophils in the injured lungs of cystic fibrosis (CF) and acute respiratory distress syndrome (ARDS). A heparin derivative, 2-O, 3-O desulfated heparin (ODSH), has been shown to inhibit HMGB1 release from a macrophage cell line and is efficacious in increasing bacterial clearance in a mouse model of pneumonia. Thus, we hypothesized that ODSH can attenuate the bacterial burden and inflammatory lung injury in CF and we conducted experiments to determine the underlying mechanisms.

**Methods:**

We determined the effects of ODSH on lung injury produced by *Pseudomonas aeruginosa* (PA) infection in CF mice with the transmembrane conductance regulator gene knockout (*CFTR*^*−/−*^). Mice were given ODSH or normal saline intraperitoneally, followed by the determination of the bacterial load and lung injury in the airways and lung tissues. ODSH binding to HMGB1 was determined using surface plasmon resonance and in silico docking analysis of the interaction of the pentasaccharide form of ODSH with HMGB1.

**Results:**

CF mice given 25 mg/kg i.p. of ODSH had significantly lower PA-induced lung injury compared to mice given vehicle alone. The CF mice infected with PA had decreased levels of nitric oxide (NO), increased levels of airway HMGB1 and HMGB1-impaired macrophage phagocytic function. ODSH partially attenuated the PA-induced alteration in the levels of NO and airway HMGB1 in CF mice. In addition, ODSH reversed HMGB1-impaired macrophage phagocytic function. These effects of ODSH subsequently decreased the bacterial burden in the CF lungs. In a surface plasmon resonance assay, ODSH interacted with HMGB1 with high affinity (K_D_ = 3.89 × 10^–8^ M) and induced conformational changes that may decrease HMGB1’s binding to its membrane receptors, thus attenuating HMGB1-induced macrophage dysfunction.

**Conclusions:**

The results suggest that ODSH can significantly decrease bacterial infection-induced lung injury in CF mice by decreasing both HMGB1-mediated impairment of macrophage function and the interaction of HMGB1 with membrane receptors. Thus, ODSH could represent a novel approach for treating CF and ARDS patients that have HMGB1-mediated lung injury.

**Graphic abstract:**

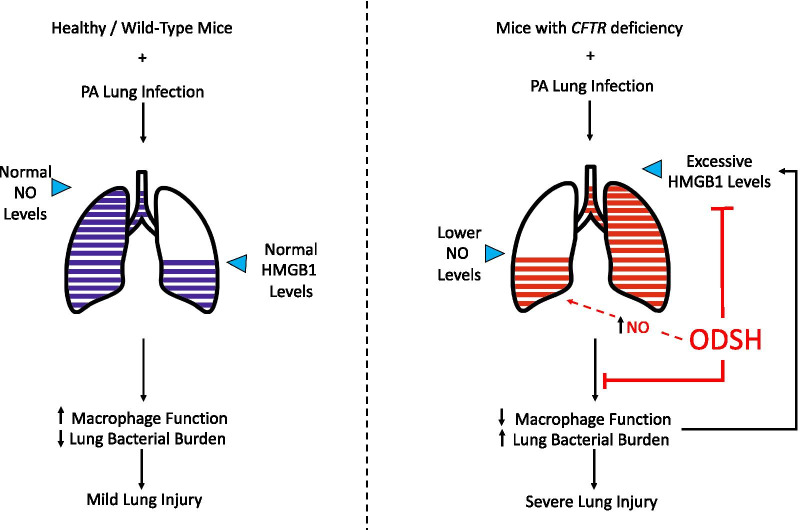

**Supplementary Information:**

The online version contains supplementary material available at 10.1186/s10020-021-00334-y.

## Introduction

Cystic fibrosis (CF) is a fatal autosomal-recessive disease caused by mutations in the gene encoding the cystic fibrosis transmembrane conductance regulator (CFTR) chloride ion channel, resulting in dysfunctional salt and water transport across the epithelia (Elborn 2016). CF is, in part, characterized by persistent lung inflammation, accumulation of thickened airway mucus and chronic bacterial infections. Gram-negative bacteria, such as *Pseudomonas aeruginosa* (PA), are commonly found in the lungs of adult CF patients (Langan et al. 2015; Parkins and Floto 2015).

Nitric oxide (NO), released by upper airway epithelial cells in response to microbial pathogens (Carey et al. 2016; Workman et al. 2017), plays an essential role in the innate immune response against bacterial infections. NO has efficacy as an antimicrobial reactive nitrogen species (RNS) and it modulates the microbicidal functions of innate immune cells (Bogdan 2015; Gore et al. 2020; Wink et al. 2011). NO is essential for the clearance of PA bacterial infection (Webert et al. 2000) but production of NO is decreased in cells and lungs of CF patients (Downey and Elborn 2000; Kelley and Drumm 1998).

Functional resident alveolar macrophages (AM), which are part of the first-line immune response, are also critical in controlling invading pathogens (Riches and Martin 2018). The phagocytosis and killing of bacteria is critical for the effective clearance of invading PA (Aberdein et al. 2013; Aderem and Underhill 1999). However, in CF patients, there is an impairment in the bactericidal efficacy of alveolar macrophages (Bessich et al. 2013; Porto et al. 2011). In addition, airway colonization with antimicrobial-resistant bacteria produces more pronounced and persistent PA infections in CF patients (Langan et al. 2015; Patel et al. 2013).

The local lung immunomodulatory environment also plays a critical role in regulating macrophage functions and bacterial clearance (Khoury et al. 2017). We and other research groups have reported that CF patients have high levels of high mobility group box 1 (HMGB1) in the sputum and bronchoalveolar lavage fluid (BALF) (Entezari et al. 2012; Griffin et al. 2013; Liou et al. 2012; Rowe et al. 2008). HMGB1 is a nuclear protein that is passively released by injured cells or actively secreted by immune cells such as macrophages (Chen et al. 2013; Lu et al. 2014; Willenbrock et al. 2012). In healthy individuals, HMGB1 is released into the extracellular environment, where it functions as damage-associated molecular pattern (DAMP) molecule that activates specific immune cells for host defense and tissue repair (Wang et al. 2019). However, the excessive accumulation of extracellular HMGB1 seen in CF patients can decrease macrophage clearance of invading bacteria and apoptotic neutrophils (Entezari et al. 2012; Liu et al. 2008). Notably, the systemic administration of anti-HMGB1 antibodies in animal models significantly increases macrophage phagocytosis and clearance of PA lung infection and decreases the severity of lung injury (Entezari et al. 2012; Patel et al. 2013). Overall, these studies indicate that HMGB1 plays an important role in suppressing macrophage function, leading to an impaired host response toward invading pathogens (Wang et al. 2019).

Extracellular HMGB1 interacts with the receptor for advanced glycation end products (RAGE), toll-like receptors (TLRs) 2, 4 and 9, and macrophage-1 antigen (Mac-1), activating macrophages and endothelial cells, to produce and release proinflammatory mediators (Harris et al. 2012; Wang et al. 2019), such as TNF-α, interleukin-1β (IL-1β), and HMGB1 (Wang et al. 2019). Previously, we have reported that that the administration of the heparin derivative, 2-O, 3-O desulfated heparin (ODSH), which has a low anticoagulant efficacy (Fryer et al. 1997), inhibits the binding of HMGB1 to TLR2 and TLR4 and increases the clearance of PA infection in the lungs of C57BL/6 wild-type (WT) mice (Sharma et al. 2014). In this study, we sought to determine if ODSH could increase bacterial clearance and decrease lung injury in CFTR-deficient mice. We focused our study on two major mechanisms of host defense in the lung: (1) the production of NO and (2) the immunomodulatory functions of extracellular HMGB1. We also performed surface plasmon resonance experiments to delineate how ODSH interacts with HMGB1.

## Methods

### Special reagents

ODSH (2-O, 3-O-desulfated heparin) was prepared in powder form by Scientific Protein Laboratories (Waunakee, WI) (Fryer et al. 1997). The stock solution was made in distilled water to obtain a concentration of 50 mg/ml for the in vitro experiments and 20 mg/kg for the in vivo experiments. The stock solution was further diluted to intended concentrations with either normal saline for in vivo studies or cell culture media for in vitro experiments. Recombinant HMGB1 (in the reduced form) was purchased from R&D Systems (Minneapolis, MN). Published studies suggest that rHMGB1 is in a fully reduced state and can bind to RAGE, TLR2 and TLR4, to induce inflammatory responses (Entezari et al. 2014; Wang et al. 2019). Thus, the rHMGB1 used in this study was anticipated to be in the reduced form.

### Cell culture

The murine macrophage cell line, RAW 264.7, was obtained from American Type Cell Culture (ATCC, Manassas, VA), cultured in DMEM (ATCC, Manassas, VA) and supplemented with 10% FBS (Atlanta Biologicals, Flowery Branch, GA) and 100 units/mL penicillin/streptomycin in T75 flasks (Cellgro, Manassas, VA) in an incubator (Sheldon, Cornelius, OR) at 37 °C with 5% CO_2_ and 95% humidity. Cell subcultures (1:6 to 1:3) were prepared by scraping the cells when confluency reached 80–90%. Cell passages from 5 to 15 were utilized for the in vitro experiments.

### Animal model

Male C57BL/6 mice and *CFTR* knockout mice (STOCK *Cftr*^tm1Unc^ Tg(FABPCFTR)1Jaw/J), 8–12 weeks of age (The Jackson Laboratory, Bar Harbor, ME), were utilized in this study, in accordance with the Institutional Animal Care and Use Committees of St. John’s University. Upon arrival at the St. John’s University Animal Care Center, all mice were housed in the animal facility for acclimatization for 5–7 days in a specific, pathogen-free environment, at 21 °C, 50% relative humidity and a 12 h light/dark cycle. All mice had ad libitum access to standard rodent food and water.

At T = 0, all mice were anaesthetized with 60 mg/kg i.p. of sodium pentobarbital (Oak Pharmaceuticals, Lake Forest, IL), followed by inoculation with *Pseudomonas aeruginosa* at 1 × 10^7^ CFUs into the trachea through a 1 cm incision on the neck. Mice were randomized to receive two doses of normal saline, 8.3 or 25 mg/kg i.p. of ODSH at T = 0 and 12 h. Eighteen hours after bacterial inoculation (T = 18 h), all mice were euthanized with the cocktail of 100 mg/kg i.p. of ketamine and 10 mg/kg i.p. of xylazine in normal saline. Bronchoalveolar lavage fluid (BALF), lung homogenate and tissues were collected using cardiopuncture, as described previously (Patel et al. 2013).

### Bronchoalveolar lavage

Mice were euthanized with the cocktail of 100 mg/kg i.p. of ketamine and 10 mg/kg i.p. of xylazine in normal saline. A 1 cm incision was made on the neck to expose the trachea. A 20-gauge × 1.25-inch intravenous catheter was inserted into the trachea caudally to allow for the gentle lavage of the lungs twice with 1 mL of sterile and nonpyrogenic PBS. Two mL BALF samples were centrifuged at 1100 rpm at 4 °C for 10 min to separate the cells and lavage fluid. Supernatant samples were further assayed to determine HMGB1 levels and total protein levels using western blotting and the bicinchoninic acid assay (BCA), respectively, and were stored at − 80 °C. The cells were further assayed to determine the number of various immune cells using cytospin. The bacterial count in the BALF was determined using the colony assay.

### Differentiation of bone marrow-derived macrophages (BMDM)

Bone marrow-derived macrophages (BMDMs) were differentiated using L929 cell-conditioned media (LCM). LCM was created by using fibroblast NCTC clone 929 cells (L929 cells, ATCC, Manassas, VA) that were cultured in RPMI 1640 media (Corning Cellgro, Manassas, VA) supplemented with 10% FBS, at an initial confluence of 50% in T75 flasks for 5 days. The conditioned media was collected and filtered through a 0.22 µm filter and was stored in 50 mL aliquots at − 20 °C. Both femurs from WT and *CFTR*^*−/−*^ mice were collected and bone marrow was flushed out with LCM into a 15 mL falcon tube. BMDM cells were cultured in T75 flasks in RPMI 1640 media supplemented with 10% FBS, 1% penicillin and streptomycin and 10% LCM. The cells were allowed to grow and differentiate for 7 days and the media was changed every other day. BMDM were then collected from the flasks by gentle scraping. The cells were cultured in 24-well plates using completed RPMI media for 24 h, before performing the phagocytosis assay.

### Isolation of alveolar macrophages (AM)

Mice were euthanized with 100 mg/kg i.p. of ketamine and 10 mg/kg i.p. of xylazine in normal saline. A 1–2 cm incision was made on the neck, the trachea was exposed and a 20-gauge × 1-inch intravenous catheter was inserted caudally into the lumen of the trachea. The lungs were gently washed three times with 1 mL of pre-warmed sterile PBS with 0.5 mM of EDTA. The collected BALF was centrifuged for 10 min at 1100 rpm at 4 °C. The cell pellets were suspended in 1 mL of DMEM media (American Type Culture Collection, Manassas, VA) supplemented with 10% fetal bovine serum (Atlanta Biologicals, Lawrenceville, GA), 1% penicillin and 1% streptomycin (Life Technologies, Grand Island, NY) and cultured in 24-well plates with 1 × 10^5^ cells/well for 24 h at 37 °C with 5% CO_2_.

### Lung tissue histology

The lungs from each mouse were instilled with a 10% neutral buffered formalin solution at 20 cm of column pressure through a 20-gauge angiocatheter from the trachea. The inflated lungs were ligated and removed and stored in neutral buffered formalin solution overnight, and placed in paraffin as previously described (Entezari et al. 2014). Lung sections were prepared, stained using hematoxylin and eosin and histopathology scores were determined using an Evos XL core microscope (Life Technologies, Grand Island, NY). Histopathological analysis was performed by blinded independent investigators using a previously described lung injury scoring system (Szarka et al. 1997). The evaluation of each group was done using 10–14 mice and each mouse was scored based on 4 random fields on microscopic images by two trained lab members that were blind as to the group identity.

### Western blotting

The levels of airways HMGB1 in BALF samples and extracellular HMGB1 in cell culture media were determined using western blot or an automated capillary-based Western blot system, WES (ProteinSimple, San Jose, CA), according to manufacturer’s protocol. For conventional western blotting, equal volumes of BALF samples or cell culture samples were loaded on to 17% SDS gels and transferred to PVDF membranes. The membranes were blocked with 5% dry milk (Bio-Rad, Hercules, CA) in TBS containing 0.1% Tween 20 (TBST) for 1 h at room temperature. After three rinses with TBST, the membranes were incubated with rabbit anti-HMGB1 polyclonal antibody (Sigma Aldrich, St. Louis, MO) at a 1:1000 dilution overnight at 4 °C. The membranes were washed three times with TBST and incubated with anti-rabbit horseradish peroxide–coupled secondary antibody (1:5000; GE Healthcare, Piscataway, NJ) for 1 h at room temperature. The protein bands on the membranes were visualized using an enhanced chemiluminescence reagent kit (GE Healthcare Bio-Sciences, Pittsburgh, PA) and a BioSpectrum 600 Imaging system (UVP, Upland, CA) and the relative intensity of the bands were determined by ImageJ software (NIH, Rockville, MD). During WES, rabbit anti-HMGB1 polyclonal antibody (Sigma Aldrich, St. Louis, MO) was used at a 1:100 ratio as per the database on the manufacturer’s website.

### Phagocytosis assay

The phagocytosis assay was performed as previously described (Entezari et al. 2012), with minor modifications. RAW 264.7 cells, AM and BMDM were seeded in 24-well plates at a density of 5 × 10^4^ cells/well and were allowed to adhere overnight in an incubator. The cells were incubated with 1, 10 or 100 µg/mL of ODSH for 24 h and the phagocytosis assay was conducted. During the phagocytosis assay, the cells were incubated with FITC-labeled latex minibeads (Polysciences, Warrington, PA) that were opsonized in FBS for 1 h, at a cell/beads ratio of 1:100. The macrophages were then rinsed with cold PBS, fixed with 4% paraformaldehyde for 10 min and stained with 14.3 µM DAPI (Sigma-Aldrich, St. Louis, MO) and Rhodamine Phalloidin (Life Technologies, Grand Island, NY), to visualize the nucleus and the cytoplasm, respectively. Phagocytosis was assessed using immunofluorescent microscopy (Nikon, Melville, NY; ImageJ, NIH, Rockville, MD) and the results were expressed as the percentage of the average number of phagocytosed beads in each group compared to the control group, where approximately 200 cells from each group were quantified using ImageJ software (NIH, Rockville, MD).

### Griess assay

A modified Griess assay was performed to determine the nitrate and nitrite levels in cell lysate samples and lung tissues. A 100 µM standard solution of sodium nitrite was serially diluted and concentrations from 1.6 to 100 µM of the standard were measured in duplicate to prepare the standard curve. One hundred µL of each sample was loaded, in duplicate, on the plates, followed by addition of 100 µL of VCl_3_ and freshly prepared Griess reagents 50 μL sulfanilamide (SULF) and 50 μL of *N*-(1-naphthyl)ethylenediamine dihydrochloride (NEDD). The samples were incubated for 30 min at room temperature and the absorbance was measured at 450 nm using a spectrophotometry with a plate reader and Ascent software (Thermo Scientific, Rockford, IL) and the NOx levels were calculated based on the standard curve.

### Surface plasmon resonance

The interaction of ODSH with HMGB1 was performed using a gold nanoparticle-based localized surface plasmon resonance (SPR) (Open SPR, Nicoya Life Systems, Kitchener, ON, Canada). HMGB1 was bound to the amine sensor chip (Cat#SEN-Au-100-10-AMINE), according to the manufacturer’s instructions and after obtaining a stable baseline, various concentrations of ODSH were placed in the running buffer and passed over the sensor chip. The resulting spectrogram was analyzed by Trace Drawer Kinetic Data Analysis v.1.6.1 (Nicoya Life Systems), using a one-to-one model (i.e. one monovalent ligand binding to one target).

### Molecular docking

To determine the binding interactions of ODSH with HMGB1, the 3D protein file of HMGB1, consisting of protein structure determined by NMR (pdb id: 2YRQ), was downloaded from rcsb.org and imported into Schrödinger Maestro V. 2016-4 software on a Mac Pro 6-core workstation consisting of Intel Xenon X5 processor. The imported protein was then subjected to energy minimization using the Protein Preparation Wizard tool [2016-4 S.R. Epik; Impact; and Prime (Schrodinger LLC, New York, 2016)], using default options and a disulfide bond between Cys30 and Cys52 was generated and the energy minimization step was repeated using the same tool with default options. Amino acid residues 96–115 were chosen for grid generation as these were reported to produce an inflammatory response similar to HMGB1 (Yang et al. 2010) and thus was used to generate the grid for docking ODSH with HMGB1, using Glide Receptor Grid Generation tool (Glide, Schrödinger, LLC, New York, NY, 2016), with the default options. The structure of ODSH was downloaded from PubChem and refined using Ligand Preparation tool (LigPrep, Schrödinger, LLC, New York, NY, 2016) after minimizing the structure to a pentasaccharide form due to computational limitations. Molecular docking was initiated using an induced-fit docking protocol between the pentasaccharide form of ODSH and HMGB1 using default parameters (Maestro and Glide/Prime, Schrödinger, LLC, New York, NY, 2016). Docking scores with different conformations of ODSH bound HMGB1 were generated and evaluated by visual inspection.

### Statistics

Data for multiple comparisons were analyzed using one-way ANOVA and post hoc analysis was done using Dunnett’s test or Tukey’s test. Data for comparing two groups, such as the release of NO upon exposure to PA and macrophage phagocytic activities, were analyzed using unpaired Student’s *t* test. The results are presented as the mean ± SEM of at least three independent experiments. The statistical analysis was done using GraphPad Prism statistical software (La Jolla, CA). A confidence interval of 95% and a p value < 0.05 was considered statistically significant.

### Animal study approval

The animal protocol for mice used in this study was in accordance with the federal and state regulations regarding the care and use of laboratory animals and the NIH Guide for the Care and Use of Laboratory Animals and approved by the St John’s University IACUC.

## Results

### ODSH decreases lung injury in CF mice with PA infection

**Fig. 1:**
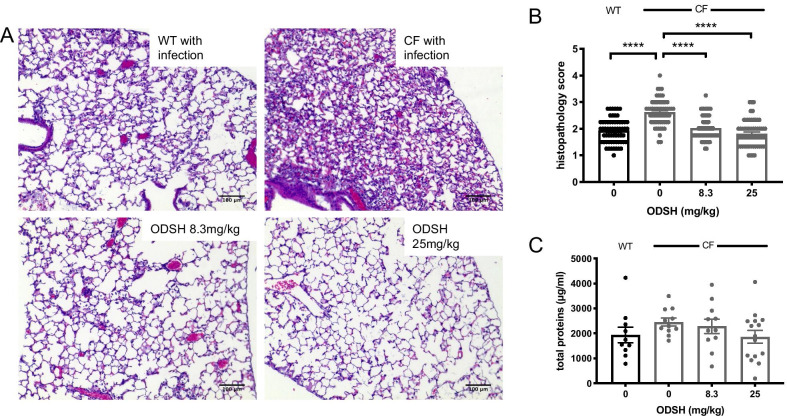
ODSH attenuates lung injury in CF with PA infection. C57BL/6 mice (WT) and *CFTR*^*−/−*^ mice (CF) were intratracheally inoculated with Pseudomonas aeruginosa (PA) at T = 0 and were randomized to receive via i.p. administration of saline or ODSH at 8.3 or 25 mg/kg at T = 0 and T = 12 h. 18 h post PA inoculation (T = 18 h), mouse lungs were perfused with 4% formaldehyde and harvested from the mice, followed by paraffinization, tissue sectioning and H&E staining. **A** Histological analysis was performed and the **B** level of lung injury was scored. **C** Bronchoalveolar lavage fluid (BAL) was collected 18 h after infection and was analyzed for total protein content by bicinchoninic acid assay (BCA), which serves as a marker of lung injury. Data represent mean ± SEM of three independent experiments (n = 10–14 mice) for each group. One-way ANOVA was used for multiple group comparison to determine statistical significance between control CF group and other groups. ****, P < 0.0001 compared to the control CF mice administered with saline

To determine if ODSH has protective effects on PA infection-induced inflammatory lung injury exacerbated by CF deficiency, *CFTR*^*−/−*^ (CF) mice were intratracheally inoculated with 1 × 10^7^ CFUs of PA at T = 0. These mice were then treated with 8.3 or 25 mg/kg i.p. of ODSH, or saline (vehicle control) at T = 0 and 12 h post PA inoculation (T = 12 h). After 18 h (T = 18 h), the lungs were either fixed and subjected to histopathological analysis for lung injury, or lavaged to collect BALF. As shown in the histological images (Fig. 1A), the lungs of CF mice infected with PA had more thickened alveolar walls and greater proteinaceous debris along the alveolar walls, compared to the lungs of WT mice that received the same inoculum of PA. Comparatively, CF mice infected with PA and treated with ODSH had clearer alveolar spaces, with less thickening of the alveolar walls. These histopathological changes were further quantified for alveolar wall thickening, infiltration of inflammatory cells and deposit of proteinaceous debris in the lining of the alveolar duct and alveolar space, as described by a histopathological scoring system of lung injury (Szarka et al. 1997). CF mice treated with saline vehicle (Fig. 1B) had significantly higher levels of inflammatory lung injury compared to WT mice (2.621 ± 0.062 of CF versus 1.927 ± 0.059 histopathological score of WT, p < 0.0001).

The administration of 8.3 mg/kg (2.020 ± 0.056 histopathological score, p < 0.0001) and 25 mg/kg (1.817 ± 0.063 histopathological score, p < 0.0001) i.p. of ODSH to CF mice significantly decreased inflammatory lung injury, compared to CF mice treated with vehicle (Fig. 1B). In addition, the BALF from CF mice contained higher mean levels of protein (2454 ± 159.1 µg protein/mL) compared to WT mice (1934 ± 309.7 µg protein/mL), although this difference was not statistically significant (p = 0.4029). In CF mice treated with 8.3 mg/kg (2281 ± 283.6 µg protein/mL, p = 0.9395) and 25 mg/kg i.p. of ODSH (1859 ± 254.8 µg protein/mL, p = 0.2292), there was a non-significant decreasing trend in the levels of airway proteins, compared to CF mice treated with vehicle (Fig. 1C). The total protein content in BALF of CF mice treated with 25 mg/kg i.p. of ODSH (1858 ± 254.8 µg protein/mL) was decreased to a similar level as that of the WT mice (1934 ± 309.7 µg protein/mL). These data suggest that ODSH decreases PA-induced inflammatory lung injury in CF mice.

### ODSH decreases the increased bacterial burden in PA-infected CF mice

**Fig. 2:**
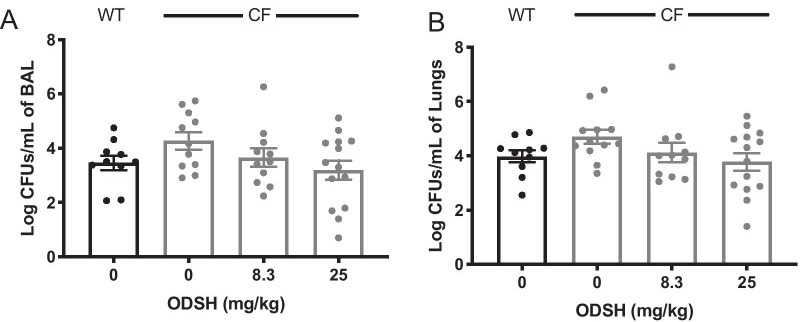
ODSH improves bacterial clearance in BALF and lung tissues of CF mice. C57BL/6 mice (WT) and *CFTR*^*−/−*^ mice (CF) were intratracheally inoculated with Pseudomonas aeruginosa (PA) at T = 0 and were randomized to receive via i.p. administration of saline or ODSH at 8.3 or 25 mg/kg at T = 0 and 12 h.  **A** Bronchoalveolar lavage fluid (BALF) and **B** lung homogenate were harvested 18 h after infection (T = 18 h) and were analyzed for the amount of viable bacteria by plating serial dilutions of BALF samples followed by colony assay. Bacterial load in the samples is expressed as log CFUs/mL in BALF or lungs. Data represent the mean ± SEM of four independent experiments (n = 10–14). One-way ANOVA was used for multiple group comparison to determine statistical significance between control CF group and other groups

To determine if ODSH’s decrease in lung injury in CF mice with PA lung infection was due to a decrease in the bacterial burden, we performed quantitative bacteriology on lung tissue homogenate and the BALF as previously described (Entezari et al. 2012). We have reported that the bacterial burden is significantly increased in CF mice compared to WT mice (Entezari et al. 2012). Similar results were obtained in the CF and WT mice, as shown in Fig. 2. The CF mice had higher bacterial loads in the BALF (4.271 ± 0.318 log CFUs/mL) and the lung tissues (4.699 ± 0.260 log CFUs/mL), compared to the WT control BALF (3.461 ± 0.268 log CFU/mL) and lung tissues (3.980 ± 0.222 log CFUs/mL) (Fig. 2A and B). Although it is not statistically significant, both 8.3 and 25 mg/kg i.p. of ODSH decreased the bacterial burden in the BALF (CF control: 4.271 ± 0.318 CFUs/mL; 8.3 mg/kg: 3.659 ± 0.335 log CFUs/mL; 25 mg/kg: 3.187 ± 0.360 CFUs/mL) and lung tissues (CF control: 4.699 ± 0.260 CFUs/mL; 8.3 mg/kg: 4.125 ± 0.359 log CFUs/mL; 25 mg/kg: 3.778 ± 0.320 log CFUs/mL), compared to the CF control group. The mean bacterial burden in CF mice treated with 25 mg/kg i.p. of ODSH were similar or even lower, compared to WT mice in the BALF (3.187 ± 0.360 CFUs/mL in CF mice vs 3.461 ± 0.268 log CFU/mL in WT mice) and lung tissues (3.778 ± 0.320 log CFUs/mL in CF mice vs 3.980 ± 0.222 log CFUs/mL in WT mice). These results suggest that high bacterial burden in airways and lung tissues of CF mice can be decreased by ODSH to levels similar to those in mice without a deficiency in CFTR.

### ODSH ameliorates the reduced nitrate levels in PA infected CF mice

**Fig. 3:**
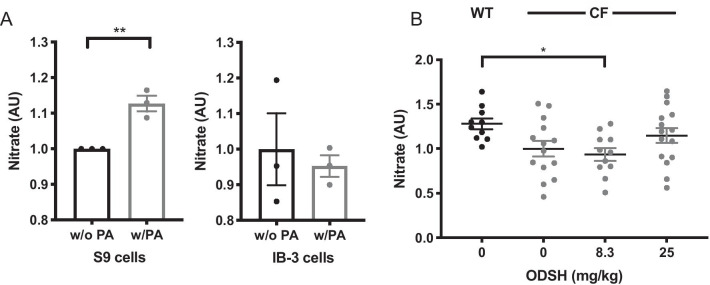
ODSH increases the reduced nitrate levels in PA infected CF mice. **A** CFTR mutated bronchial epithelial IB3-1 cells and CFTR corrected S9 control epithelial cells were cultured with or without heat killed *Pseudomonas aeruginosa* (PA) at 100 µg/mL for 24 h. Cells supernatant were collected and analyzed for the levels of extracellular nitric oxide (NO) through measuring the levels of nitrate by Griess assay. Levels of nitrate were represented by relative optical density and expressed in AU. Each value represents mean ± SEM of three independent experiments for each group. **, P < 0.01 compared to the S9 control cell group infected with PA. **B** C57BL/6 mice (WT) and *CFTR*^*−/−*^ mice (CF) were intratracheally inoculated with Pseudomonas aeruginosa (PA) at T = 0 and were randomized to receive via i.p. administration of saline or ODSH at 8.3 or 25 mg/kg at T = 0 and 12 h. Bronchoalveolar lavage fluid (BALF) was collected 18 h after infection (T = 18 h) and the levels of NO were analyzed by measuring the levels of nitrate by Griess assay. Values (n = 10–14 mice) were represented by relative optical density and expressed in AU. Each value represents mean ± SEM of three independent experiments for each group. One-way ANOVA was used for multiple group comparison and unpaired t test was used for two group comparison to determine statistical significance

NO is important in bacterial killing and its production by lung cells, including epithelial cells, must be increased in order to effectively clear invading pathogens in the lungs (Darling and Evans 2003). However, NO levels are lower in the exhaled breath from CF patients compared to healthy individuals (Malerba et al. 2014). We first determined the secretion of NO from CF following their exposure to heat-killed PA, using the CFTR deficient epithelial cell line, IB3-1 cells. Although the nitrate levels were significantly increased in the media of S9 cells exposed to PA (1.13 ± 0.02 versus 1 AU, p < 0.01), the mean levels of NO produced by IB3-1 cells in the presence of heat-killed PA were comparable or even lower than the basal levels of NO (0.95 ± 0.03 AU) (Fig. 3A). These data suggest that NO production is decreased in CF epithelial cells exposed to PA infection. We then determined if CF mouse lungs also had low levels of NO after PA infection. The basal level of NO in the lung tissues of CF mice (1.000 ± 0.087 AU) was lower than that of WT mice (1.28 ± 0.060 AU) upon PA challenge. These data suggest that the decreased secretion of NO in response to PA infection by CF epithelial cells may contribute to the decreased NO levels in the lungs of CF patients.

To further evaluate whether ODSH-enhanced bacterial clearance in CF mice is due to the restoration of NO levels in CF mice following PA infection, we measured lung NO levels in CF mice treated with either vehicle or different doses of ODSH. ODSH, at 8.3 mg/kg i.p., did not reverse, but further decreased lung NO levels (0.934 ± 0.071 AU), compared to CF mice treated with vehicle (1.000 ± 0.087 AU, p = 0.097). In contrast, the lung NO levels in mice treated with 25 mg/kg i.p. of ODSH were increased (1.148 ± 0.084 AU), though not significantly. The NO level in the mice treated with 8.3 mg/kg i.p. of ODSH was significantly lower than WT mice treated with saline (0.934 ± 0.071 of ODSH low dose group versus 1.280 ± 0.060 of WT control, p < 0.05), whereas the NO levels in mice treated with 25 mg/kg i.p. of ODSH was comparable to WT mice treated with saline (1.148 ± 0.084 AU of ODSH high dose versus 1.280 ± 0.060 AU of WT control). (Fig. 3B). These results suggest ODSH not only partially restores the decreased NO secretion following PA infection in CF mice but it may also affect additional mechanisms to restore the bacterial clearance in the lungs of the CF mice.

### Alveolar macrophages from CF mice have a greater impairment in  phagocytosis than macrophages derived from bone marrow

**Fig. 4:**
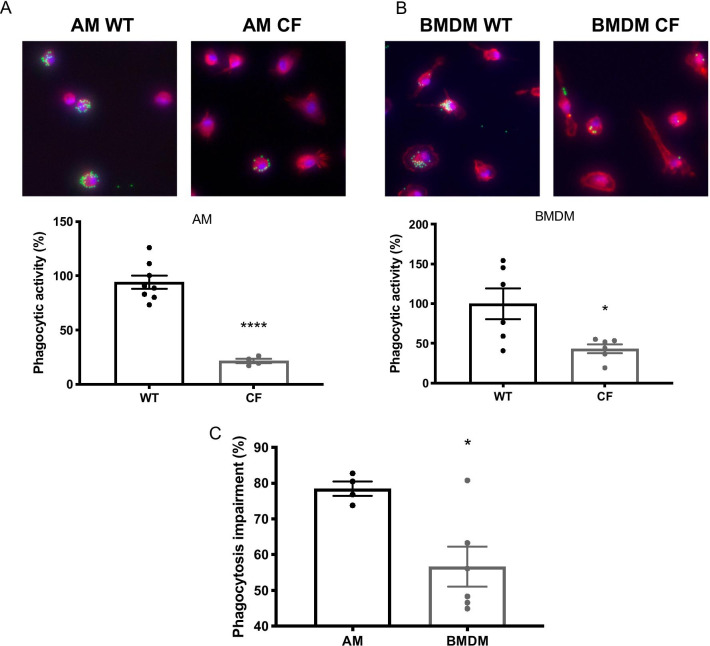
Phagocytic function is compromised in macrophages isolated from CF mice. Bone marrow derived macrophages (BMDM) and alveolar macrophages (AM) isolated from C57BL/6 mice (WT, n = 3 mice) and *CFTR*^*−/−*^ mice (CF, n = 3 mice) were cultured. Phagocytosis assay was performed on **A.** AM and **B.** BMDM by incubating cells with FITC-labeled latex mini-beads (green stain) for 1 h and then staining with DAPI (blue nuclear stain) and phalloidin (red cytoskeletal stain) to visualize the amount of phagocytosed beads per cell. Fluorescent micrographs were quantified and the phagocytic ability of macrophages was represented as a percent phagocytic activity normalized to WT control group. Each value represents mean ± SEM of three independent experiments for each group. *, P < 0.05, ****, P < 0.0001, compared to control groups. **C** To determine if the magnitude of phagocytosis impairment in CF macrophages is similar between the alveoli and bone marrow macrophages, the phagocytosed beads for AM and BMDM isolated from CF mice were compared to their WT counterparts and expressed as the percent of phagocytosis impairment of the WT macrophages. Each value represents mean ± SEM of three independent experiments for each group. *, P < 0.05 compared to AM group. Unpaired t-test was used for comparison between two groups

Previous studies have reported a deficit in the clearance of invading bacterial pathogens, such as PA, in the lungs of CF patients (Darling and Evans 2003; Langan et al. 2015). To determine whether CFTR deficiency impaired macrophage function, we determined the phagocytic activity of macrophages from CFTR deficient mice. Both bone marrow-derived macrophages (BMDM) (43.34 ± 5.58%, p < 0.05) and alveolar macrophages (AM) (21.56 ± 1.98%, p < 0.0001) harvested from CF mice had a significantly lower phagocytic activity, compared to the BMDM control (100.0 ± 19.41%) and AMs (94.20 ± 6.171%) from WT mice (Figs. 4A and B). The magnitude of phagocytosis impairment in AM and BMDM was expressed as percentage change and the impairment of AMs in the CF mice was greater than that of CF BMDM (phagocytosis impairment as 78.44 ± 1.985% vs 56.66 ± 5.584%, p < 0.05) (Fig. 4C). These results suggest that although CFTR deficiency can directly impair phagocytic activity, this effect can be further exacerbated by the airway microenvironment.

### ODSH decreases the airway levels of HMGB1 in PA-infected CF mice

**Fig. 5:**
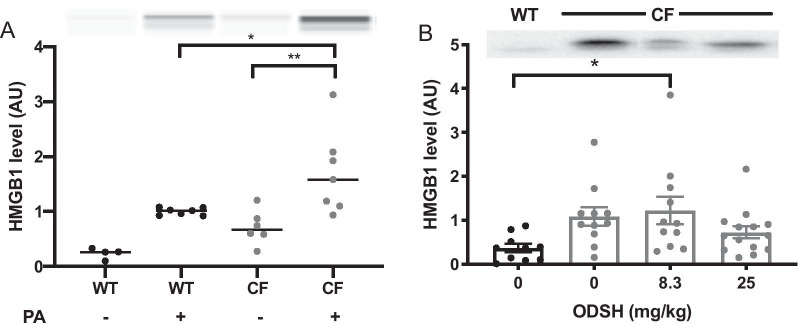
ODSH decreases airway HMGB1 accumulation in PA infected CF mice. **A** C57BL/6 mice (WT) and *CFTR*^*−/−*^ mice (CF) were randomized and inoculated with normal saline (without PA) or PA (1 × 10^7^ CFUs/mouse) for 18 h. Bronchoalveolar lavage fluid (BALF) was collected from mice was analyzed for the levels of extracellular HMGB1 by Immunoblot blot analyses. Representative images of immunoreactive bands of HMGB1 in the samples were shown on the top of the bar graphs. **B** C57BL/6 mice (WT) and *CFTR*^*−/−*^ mice (CF) were intratracheally inoculated with Pseudomonas aeruginosa (PA) at T = 0 and were randomized to receive via i.p. administration of saline or ODSH at 8.3 or 25 mg/kg ta T = 0 and 12 h. BALF was collected 18 h after infection (T = 18 h) and was analyzed for the levels of airway HMGB1 by Western blot analysis. Levels of HMGB1 were represented by relative densitometric values and expressed in AU. Each value represents mean ± SEM of three independent experiments for each group. *, P < 0.05; **, P < 0.01 compared to WT groups infected with PA (**A**). *, P < 0.05 compared to CF control groups receiving normal saline. One-way ANOVA was used for multiple group comparison to determine statistical significance

Previous studies in our lab have shown that the excessive accumulation of HMGB1 in airways of CF patients impairs bacterial clearance in mice with PA lung infections (Entezari et al. 2012). Similarly, the results of this study indicated an excessive accumulation of airway HMGB1 in CF mice compared to WT mice (Fig. 5). We therefore determined if the i.p. administration of ODSH alters HMGB1 accumulation in airways of CF mice. As shown in Fig. 5A, immunoblot analysis of airway HMGB1 levels indicated an increase in the HMGB1 level in the airways of WT mice infected with PA (1.00 ± 0.02 AU), compared to the non-PA infected WT mice (0.24 ± 0.05 AU). After PA infection, airway HMGB1 levels in CF mice were significantly increased (1.71 ± 0.29 AU, p < 0.01), compared to the non-infected control CF mice (0.71 ± 0.13 AU) (Fig. 5A). Although the difference in the levels of airway HMGB1 between CF and WT mice in the absence of PA infection was not statistically significant, after PA infection, the CF mice had significantly elevated levels of airway HMGB1 comparing to WT mice infected with PA (1.71 ± 0.29 AU in CF vs 1.00 ± 0.02 AU in WT, p < 0.05) (Fig. 5A).

In CF mice with PA lung infection, the administration of 8.3 mg/kg i.p. of ODSH did not significantly decrease airway HMGB1 levels (1.22 ± 0.31 AU), compared to CF mice treated with vehicle (1.13 ± 0.19 AU) (Fig. 5B). The airway levels of HMGB1 were significantly increased by 8.3 mg/kg i.p. of ODSH (1.22 ± 0.31 AU, p < 0.05), compared to PA-infected WT control mice (0.36 ± 0.09 AU) (Fig. 5B). At 25 mg/kg i.p., ODSH did not significantly decrease airway HMGB1 levels in PA-infected CF mice, compared to mice treated with vehicle, but did reduce the levels of airway HMGB1 to 0.72 ± 0.14 AU, which was not significantly different from those in PA-infected WT control mice (0.36 ± 0.09 AU) (Fig. 5B). These results suggest that HMGB1 accumulation in airways of PA-infected mice is exacerbated by CFTR deficiency and that ODSH, at 25 mg/kg i.p., decreases airway HMGB1 in CF mice infected with PA to a level similar to that of the WT mice. Thus, mechanisms, in addition to the decrease in airway HMGB1 levels and the increase in NO secretion, may be involved in ODSH-increased host defense against PA infection in CF mice (complete gel image is in Additional file 1: Fig. 1).

### ODSH attenuates  the impairment of HMGB1-induced macrophage phagocytosis

**Fig. 6:**
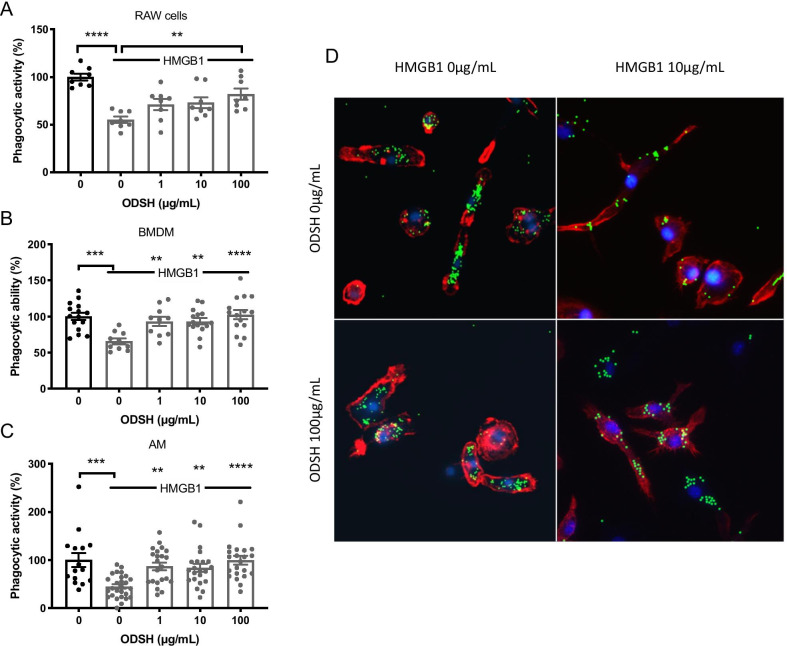
ODSH improves HMGB1-compromised phagocytosis of macrophages. Murine macrophage cells line **A** RAW 264.7 cells, and primary **B** bone-marrow derived macrophages (BMDMs), and **C** alveolar macrophages (AM) isolated from *CFTR*^*−/−*^ mice (CF, n = 3 mice) were incubated with recombinant HMGB1 (10 µg/mL) without or with co-treatment with ODSH at 1, 10, 100 µg/mL for 24 h. Phagocytosis assay was performed on the macrophages by incubating with FITC-labeled latex mini-beads (green stain) for 1 h and then staining with DAPI (blue nuclear stain) and phalloidin (red cytoskeletal stain) to visualize the amount of phagocytosed beads per cell. Fluorescent micrographs were quantified, and the phagocytic ability of macrophages was represented as a percent phagocytic activity normalized to WT control group. **D** Represented images of macrophage phagocytosis under different conditions. One-way ANOVA was used for multiple group comparison. Each value represents mean ± SEM of three independent experiments for each group. **, P < 0.01, ***, P < 0.001, ****, P < 0.0001 compared to control groups

We have previously shown that extracellular HMGB1 impairs macrophage function by activating TLR2 and TLR4 and that ODSH can block HMGB1 from interacting with TLR2 and TLR4 (Entezari et al. 2014; Sharma et al. 2014). To determine whether ODSH directly affects HMGB1 signaling efficacy, we conducted experiments to determine if ODSH increases PA clearance in CF mice by attenuating HMGB1-mediated impairment of macrophage phagocytosis. Extracellular HMGB1 significantly impaired macrophage phagocytic function in RAW 264.7 cells (55.07 ± 3.20%, p < 0.0001), primary BMDMs (65.68 ± 4.03%, p < 0.001) and AMs (44.80 ± 4.58%, p < 0.001), compared to these same macrophages incubated with vehicle (Fig. 6A–C). The incubation of RAW 264.7 cells with ODSH significantly increased macrophage phagocytic activity from 55.07 ± 3.20% to 71.10 ± 5.89% at 1 µg/mL, 73.04 ± 5.72% at 10 µg/mL, and 81.97 ± 5.84% at 100 µg/mL (p < 0.01) (Fig. 6A). In addition, ODSH significantly attenuated the HMGB1-induced decrease in BMDMs phagocytosis at 1 µg/mL (93.45 ± 6.38%, p < 0.01), 10 µg/mL (93.33 ± 4.43%, p < 0.01), and 100 µg/mL (102.8 ± 6.46%, p < 0.0001), compared to BMDMs incubated with vehicle (65.68 ± 4.03%) (Fig. 6B). In AMs, ODSH significantly attenuated the HMGB1-mediated impairment of phagocytosis at 1 µg/mL (87.04 ± 7.88%, p < 0.01), 10 µg/mL (83.88 ± 8.34%, p < 0.01), and at 100 µg/mL ODSH (99.44 ± 8.85%, p < 0.0001) compared to AMs incubated with vehicle (44.80 ± 4.59%) (Fig. 6C). Importantly, ODSH alone did not significantly alter the intrinsic phagocytic activity of BMDMs and AMs (Additional file 1: Fig. 2). Representative images of macrophage phagocytosis using immunofluorescent microscopy are presented in Fig. 6D. These data suggest that ODSH decreases the impairment of macrophage phagocytosis produced by excessive accumulation of airway HMGB1.

### The conformation of HMGB1 is altered by the binding of ODSH

**Fig. 7:**
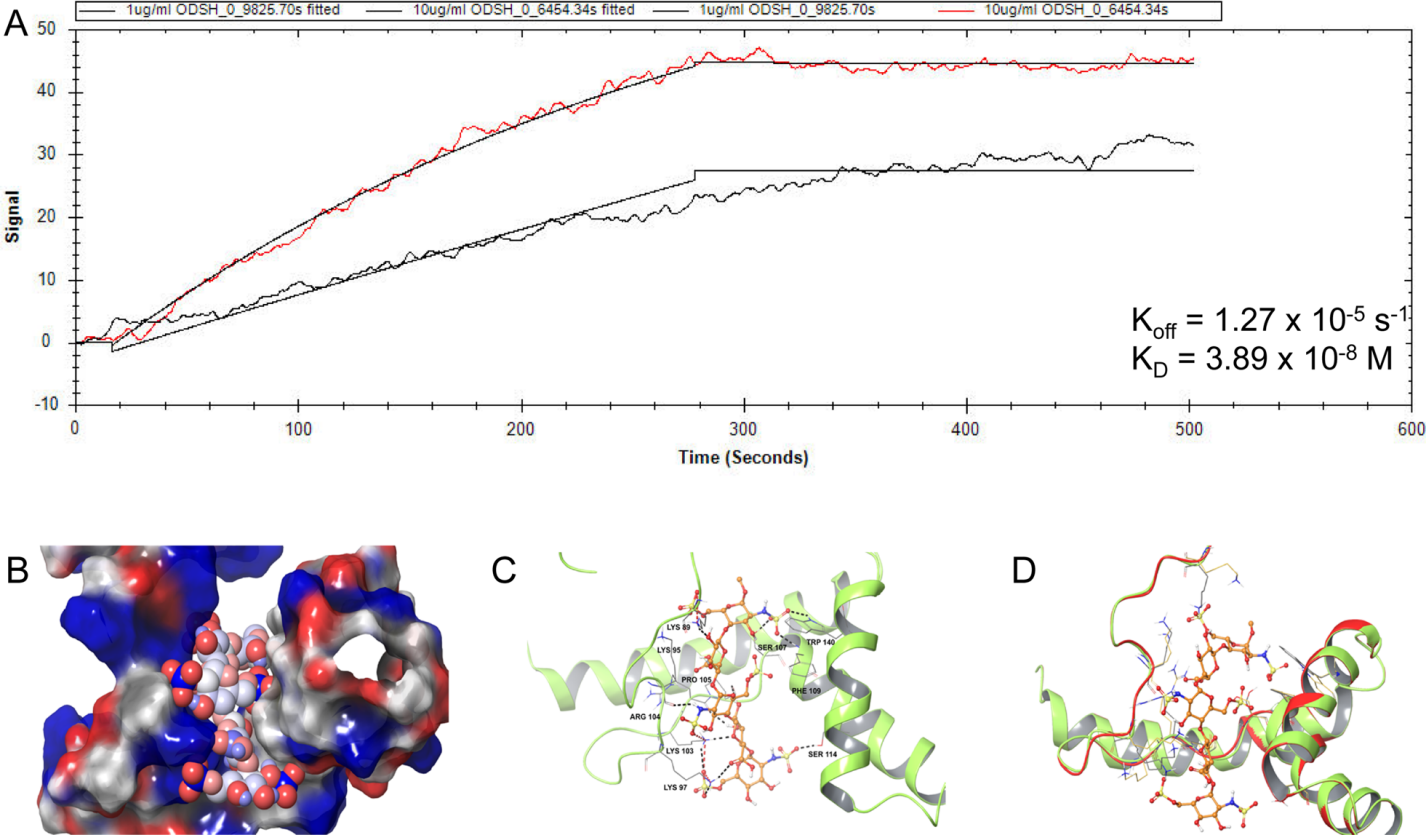
ODSH binds to HMGB1 and induces conformational changes on HMGB1. The localized surface plasmon resonance (LSPR) binding efficiency of ODSH to HMGB1 was determined by applying ODSH at 1 and 10 µg/mL and measuring its ability to bind to carboxyl sensor chips coated with recombinant HMGB1. **A** The representative kinetic binding curves demonstrating the ability of ODSH to associate and then dissociate off of HMGB1. The kinetic binding curves were then used to compute the bind efficiency (K_D_) for ODSH to HMGB1. Next, induced fit docking experiment was conducted to determine the interactions of ODSH with the B-Box domain of HMGB1 **B** Surface representation of HMGB1 with ODSH bound to the B-Box domain wherein the blue region represents the concentration of negatively charged, red for positively charged and grey for neutral amino acid residues. ODSH is represented in CPK form with the spheres following the same color pattern as the surface of protein. **C** The representative 3D docking pose of ODSH bound to HMGB1 B-Box domain. **D** Superimposition of native, energy minimized HMGB1 (red ribbons) and ODSH docked to HMGB1 (green ribbons). **C** and **D** ODSH was represented as ball and stick model whereas the amino acids are shown as thin tubes with atom colors shown as orange for carbons of ODSH, red for oxygen, blue for nitrogen, yellow for sulfur, and grey for carbons belonging to the amino acid residues of ODSH bound HMGB1 and yellow for carbons belonging to the amino acids of native HMGB1. Dashed black lines represent hydrogen bonds and dashed red lines represent ionic interactions between ODSH and HMGB1

We used surface plasmon resonance (SPR) to determine the binding affinity of ODSH to HMGB1. The SPR experiments indicated that ODSH was attached to HMGB1 under the flow conditions, with a binding affinity (K_D_) of 3.89 × 10^–8^ M (Fig. 7A). We next utilized molecular docking analysis to determine if ODSH induces a change in the conformation of HMGB1. In order to compensate for computational limitations, the pentasaccharide form of ODSH, derived from 2-O and 3-O modifications of the heparin pentasaccharide, was chosen to mimic the polysaccharide units of ODSH and was docked with HMGB1, using an induced fit docking mode (with flexible protein and ligand). The binding pose, with a docking score of − 8.292 kcal/mol, is shown in Fig. 7C and the sugar portion of ODSH formed several hydrogen bonding interactions with amino acid residues, Trp140, Phe109, Ser107/114, Lys89/95/97/103, Pro105 and Arg104, which are located in the binding site of HMGB1. In addition, the sulfonic acid and sulfonamide groups formed ionic interactions with the Lys89, Lys97 and Lys103 residues in HMGB1 (Fig. 7C). A surface representation of the same docking pose (Fig. 7B) shows that the acidic portion of ODSH is oriented toward the basic regions of HMGB1. The superposition of the ODSH docked with HMGB1, along with energy minimized native HMGB1, shows the conformational changes in the amino acid side chains of HMGB1 (Fig. 7D). These data suggest that ODSH binds to HMGB1 and induces conformational changes that could decrease its affinity toward its cognate receptors, TLR2, TLR4, and RAGE.

## Discussion

CF patients fail to clear bacteria from their airways due to a number of host-defense abnormalities, including proteolytic cleavage of the complement C3b receptor (CR3) from the surface of neutrophils, thereby preventing opsonin-facilitated PA phagocytosis (Tosi et al. 1990), proteolytic destruction of lactoferrin (Rogan et al. 2004), reduced airway production of the anti-bacterial compound, nitric oxide (Downey and Elborn 2000; Kelley and Drumm 1998), and excessive accumulation of extracellular HMGB1 (Chirico et al. 2015; Entezari et al. 2012). In this investigation, we validated our previous data (Entezari et al. 2012) indicating that CF mice do not effectively clear PA inoculated into the lung (Figs. 1 and 2) due to an impairment in macrophage phagocytic activity (Fig. 4). Previously, we had shown that deficient bacterial clearance and impaired macrophage phagocytosis in CF mice was mediated, in part, by an increase in the alarmin molecule, HMGB1, in the CF mouse airways, and that bacterial clearance and macrophage phagocytosis were enhanced by neutralizing anti-HMGB1 antibodies (Entezari et al. 2012). These findings are clinically relevant as HMGB1 levels are markedly elevated in the airways and the circulation of CF patients (Entezari et al. 2012; Griffin et al. 2013; Liou et al. 2012; Rowe et al. 2008). In this study, we evaluated a second and perhaps more practical therapeutic approach than anti-HMGB1 antibodies, namely, low anticoagulant ODSH.

The administration of ODSH to CF mice significantly decreased lung injury from PA pneumonia (Fig. 1) and increased PA bacterial clearance in the airways and lung tissues of CF mice (Fig. 2). ODSH also decreased the airway accumulation of HMGB1 in response to PA infection in CF mice (Fig. 5). This effect may be mediated by the inhibition of neutrophil elastase-induced stimulation of HMGB1 secretion (Griffin et al. 2013), as ODSH is an allosteric inhibitor of neutrophil elastase (Kummarapurugu et al. 2018), or by disrupting the nuclear export of HMGB1 by inhibiting the enzyme, p300 acetyltransferase (Zheng et al. 2017). In addition to decreasing airway HMGB1 levels, ODSH also decreased the HMGB1-induced impairment of macrophage phagocytosis (Fig. 6). This effect could have been due to, in part, the binding of HMGB1 to ODSH. As previously reported, HMGB1 is present in the fully reduced state in our mouse model (Entezari et al. 2014) and it can bind to RAGE, TLR2 and TLR4 receptors, which induces an inflammatory response by increasing the secretion of proinflammatory cytokines and chemokines (Wang et al. 2019). Results from the SPR and computational docking analysis  (Fig. 7) indicate that ODSH-induced conformational changes in reduced form of HMGB1 (Figs. 7C and D) which could contribute to its decreased interaction with cognate receptors, RAGE, TLR2 and TLR4. This is supported by our previous observation that the impaired macrophage phagocytosis produced by HMGB1 is primarily due to its activation of TLR4, and to a lesser extent, TLR2 (Entezari et al. 2012).

In normal hosts, bacterial infections stimulate NO production to control bacterial replication (Bogdan 2015). However, in CF patients, the production of lung NO is insufficient, despite chronic bacterial infection or during exacerbations of lung diseases (Downey and Elborn 2000; Kelley and Drumm 1998). NO levels in CF bronchial IB-3 epithelial cells are decreased compared to normal bronchial S9 epithelial cells (Fig. 3A), as indicated by the decrease in nitrate levels in the culture media. Nitrate concentrations were also significantly lower in PA infected CF mice compared to WT mice (Fig. 3B). However, the airway nitrate levels increased, although not significantly, in PA-infected CF mice treated with 25 mg/kg i.p. of ODSH, (Fig. 3B). Both heparin and non-anticoagulant heparins have been previously shown to increase NO levels by increasing the activity of nitric oxide synthase 1 (NOS1) (Kouretas et al. 1998). The mechanism by which the 25 mg/kg dose of ODSH increases NO production in CF cells and CF mice remains to be elucidated. However, this pharmacologic effect, if confirmed, may be an important supplemental host defense mechanism, in tandem with HMGB1 inhibition, for decreasing the incidence and severity of certain microbial lung infections.

**Fig. 8:**
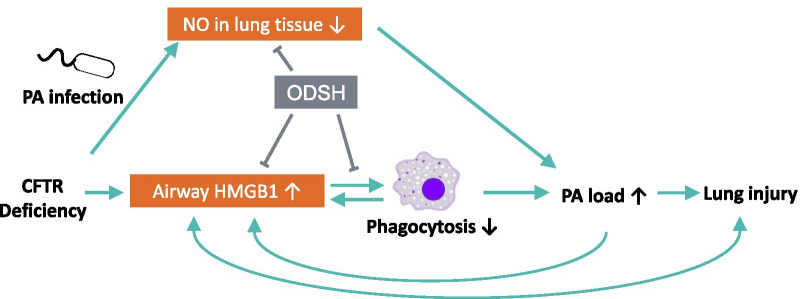
Schematic summary of the effects of ODSH on attenuating lung injury in cystic fibrosis by targeting airway HMGB1. Deficiency or dysfunction in *CFTR* gene or protein leads to an elevation of airway level of HMGB1. High levels of airway HMGB1 can compromise host defense in bacterial clearance by impairing the ability of macrophages to phagocytose the invading bacteria, resulting in increased bacterial loads in the lung and subsequently causing lung injury. Increased bacterial load in the lung and lung injury can further stimulate the release of proinflammatory mediators, including HMGB1, into the airways, establishing a deleterious cycle to cause sustained pulmonary infections and severe lung injury. Nitric oxide (NO) also plays a critical role in host defense to clear invading bacteria. However, CFTR deficiency leads to a decrease of bacterial infection-induced NO production in the lung, which can contribute to impairing host defense in clearing the invading PA. Administration of ODSH to CF mice can improve the bacterial clearing by (1) attenuating CF-suppressed NO production induced by bacterial infection in the lung, (2) decreasing the airway accumulation of extracellular HMGB1 released by necrotic cells of the injured lung as well as actively secreted by macrophages and (3) decreasing the HMGB1-mediated macrophage dysfunction. Importantly, ODSH can bind directly to HMGB1, inducing its conformational changes which might be responsible for the reduced HMGB1 binding to its receptors (e.g. TLR2 and TLR4) on macrophages. Together, ODSH protects the CF lung upon bacterial infection by attenuating HMGB1-induced innate immune dysfunction. Thus, ODSH ultimately enhances host defense in bacterial clearance by augmenting macrophage phagocytic ability, which leads to attenuating lung injury in CF

It has been postulated that certain anti-inflammatory drugs may have efficacy in the treatment of CF (Roesch et al. 2018). Currently only ibuprofen and azithromycin are approved for clinical use in CF patients and the use of anti-inflammatory drugs is not widely supported (Roesch et al. 2018). It has been suggested that the future of CF care is long-term CFTR modulator therapy for all affected patients (Bell et al. 2020). However, this poses a continued risk to CF patients due to chronic infections with antibiotic-resistant bacteria and the continued reliance on antibiotics to treat exacerbations. Thus, augmenting lung defense mechanisms could be a viable, long-term therapeutic approach for treating CF patients. Our results suggest that inhibition of the function/activity of HMGB1 by ODSH can increase lung host defense (Fig. 8) without producing the adverse effects that occur from using localized or systemic corticosteroids.

Targeted HMGB1 inhibition could also be used in non-CF patients with community acquired pneumonias. Clinical data suggest that the increased susceptibility of alcoholics to bacterial pneumonia is due, in part, to marked increases in lung HMGB1 levels (Harris et al. 2019), which have been reported to be predictive of pneumococcal bacteremia (Alpkvist et al. 2018). HMGB1 inhibition has been proposed as adjunctive therapy for pneumococcal meningitis (Masouris et al. 2017). Increased HMGB1 levels play a role in the lung injury produced by infections with *Staphylococcus aureus* (Achouiti et al. 2013) and carbapenem-resistant *Klebsiella pneumoniae* (Liming et al. 2018). Systemic corticosteroids have been shown to improve the clinical outcome in severely ill patients with community-acquired pneumonia (Torres et al. 2015) but their use is still controversial among infectious disease and critical care physicians due to their immunosuppressant effects (Waterer and Metersky 2019). Targeted anti-HMGB1 therapy with compounds, such as ODSH, could produce therapeutic efficacy without significantly altering the immune response in critically ill patients. It has been hypothesized that significant increases in the HMGB1 levels in the circulation are a primary mediator of the dysregulated inflammation in SARS-CoV2 (COVID-19) respiratory failure (Andersson et al. 2020) as increased serum HMGB1 levels are correlated with a worse outcome in COVID-19 infection (Chen et al. 2020). Subsequently, ODSH (as dociparstat sodium (DSTAT)) is currently being evaluated in a randomized double-blind clinical trial (Phase 2 and 3) for the treatment of COVID-19 pneumonia (Clinical Trial NCT04389840) (Lasky et al. 2020). ODSH treatment in PA-infected WT mice at 25 mg/kg s.q. every 12 h not only decreased lung injury and bacterial burden but also significantly decreased mortality in the absence of antibiotic treatment (Sharma et al. 2014). Adjusted for body surface area differences among species (Reagan-Shaw et al. 2008), this dose is equivalent to 4 mg/kg a day in humans, similar to those recently studied successfully in severely ill patients with acute myelogenous leukemia (Kovacsovics et al. 2018).

In the present investigation, we validated the macrophage phenotypes collected from alveoli (AM) and differentiated from bone marrow progenitor cells (BMDM) of the CF mice, both of which present compromised phagocytosis. In the optimized mouse model utilized in this study, the CFTR gene was disrupted globally, whereas the lethal problem of intestinal obstruction was corrected by expressing human CFTR in the intestines (Snouwaert et al. 1992; Zhou et al. 1994). It has been reported CFTR is expressed in phagocytic cells, such as macrophages (Bruscia and Bonfield 2016; Turton et al. 2021; Yoshimura et al. 1991), and this genetic deficiency is likely to be present in the macrophages in this study, affecting the macrophage functions. In addition to the genetic factor, the complex lung environment resulted from CFTR deficiency, including the excessive levels of cytokines (such as HMGB1 as we show in this study) and altered NO secretion upon infection (Zheng et al. 2004), could further impact the phagocytic functions of macrophages, which is what we show in this study.

This study presents a number of limitations. CF is a complex disease with more than two thousands mutations reported on *cftr* gene (Cystic Fibrosis Mutation Database; Scotet et al. 2020). Together, with the impact from different types of environmental stress, it leads to various phenotypes in patients with CF (Bareil and Bergougnoux 2020; Terlizzi et al. 2017; Zielenski, 2000). Although a number of CF animal models have been developed in the past few decades, none of them resembles all of the pathophysiology seen in CF patients. The CF mouse model used in this study displays the pathologic changes in the airways that include failure of effective pathogen clearance and hyperinflammation of alveoli (Guilbault et al. 2007; Kent et al. 1997; Semaniakou et al. 2019; Zhou et al. 1994). In addition, these CF mice do not develop spontaneous lung infections, of which the mechanisms still remain unclear. Thus, we challenged animals with PA, which is one of the most prevalent and persistent infections that occurs in CF patients despite antibiotic treatment. We have previously reported that PAO1, a laboratory strain of PA that has pathogenic characteristics similar to that of CF clinical isolates (Morrow et al. 2007), can induce moderate to severe lung injury in CF mice (Entezari et al. 2012). The qualitative and quantitative histopathological analysis of the CF lung tissues in the current study indicated that a moderate level of lung injury resulted from infection with PAO1. Our conclusions are also supported by other published studies which reported that CF mice can develop inflammatory injury due to PA infections (Guilbault et al. 2005; Heeckeren et al. 1997; Hoffmann et al. 2005). Moreover, although this mouse model of PA infection is not an ideal model that completely recapitulates the immune phenotypes of humans with CF, we believe that it can still provide insights into certain aspects of the immune responses to bacterial infections in CF, particularly the role of HMGB1 in CF pathology. We have previously reported that high levels of HMGB1 in the airways of patients with CF compromise innate immunity by decreasing airway bacteria clearance (Entezari et al. 2012). Another limitation of this study is that only murine macrophages were used. Given the data indicating the efficacy of ODSH in decreasing bacterial burden and lung injury in this CF mouse model, we will be conducting experiments to validate the results in primary macrophages from mice to those of patients with CF.

## Conclusions

The results in this study suggest that pathophysiological changes, including acute lung injury and the increased bacterial burden in the CF mouse lung, can be effectively attenuated by ODSH. The efficacy ODSH in CF mice is due, in part, to attenuating the (1) decrease in NO levels and (2) increase in the extracellular levels of HMGB1 in the airways. Furthermore, ODSH directly attenuated the HMGB1-mediated impairment of macrophage phagocytosis, thereby significantly increasing bacterial clearance in the CF mouse lung. ODSH directly binds to HMGB1, which may induce conformational changes in the conformation of HMGB and decrease its binding to its cognate receptors, thus decreasing macrophage dysfunction. Overall, the results of this study suggest that the inhibition of the bioactivity of HMGB1 by ODSH could represent a novel, targeted approach for increasing bacterial clearance in CF and non-CF related lung diseases.

## Supplementary Information

**Additional file 1: Figure 1.** Western blot immunoreactive images of airway HMGB1 in PA infected mice in the absence or presence of ODSH. **Figure 2.** ODSH does not significantly improve intrinsic phagocytic ability of macrophages in CF mice.

## Data Availability

The datasets used and/or analyzed during the current study are available from the corresponding author on reasonable request.

## Abbreviations

ODSH: 2-O, 3-O desulfated heparin
HMGB1: High mobility group box protein 1
CF: Cystic fibrosis
ARDS: Acute respiratory distress syndrome
CFTR: Cystic fibrosis transmembrane conductance regulator
SPR: Surface plasmon resonance
PA: *Pseudomonas aeruginosa*
BALF: Bronchoalveolar lavage fluid
NO: Nitric oxide
RNS: Reactive nitrogen species
DAMP: Damage-associated molecular pattern
RAGE: Receptor for advanced glycation end products
TLR: Toll-like receptor
Mac-1: Macrophage-1 antigen
IL-1β: Interleukin-1β
AM: Alveolar macrophage
BMDM: Bone marrow-derived macrophages
WT: Wild-type
BCA: Bicinchoninic acid assay
LCM: L929 cell conditioned media
TBST: Tris-buffered saline containing 0.1% Tween 20
SULF: Sulfanilamide
NEDD: *N*-(1-naphthyl)ethylenediamine dihydrochloride
K_D_: Binding affinity
CR3: Complement C3b receptor
NOS1: Nitric oxide synthase 1
DSTAT: Dociparstat sodium
