# Correction to: 2‑O, 3‑O desulfated heparin (ODSH) increases bacterial clearance and attenuates lung injury in cystic fibrosis by restoring HMGB1‑compromised macrophage function

**DOI:** 10.1186/s10020-021-00354-8

**Published:** 2021-09-03

**Authors:** Mao Wang, Alex G. Gauthier, Thomas P. Kennedy, Haichao Wang, Uday Kiran Velagapudi, Tanaji T. Talele, Mosi Lin, Jiaqi Wu, LeeAnne Daley, Xiaojing Yang, Vivek Patel, Sung Soo Mun, Charles R. Ashby, Lin L. Mantell

**Affiliations:** 1 Department of Pharmaceutical Sciences, St. John’s University College of Pharmacy and Health Sciences, Queens, NY 11439 USA; 2 Wake Forest University School of Medicine, Winston Salem, NC USA; 3 The Feinstein Institute for Medical Research, Northwell Health System, Manhasset, NY USA

## Correction to: Mol Med (2021) 27:79 10.1186/s10020-021-00334-y

Following publication of the original article (Wang et al. 2021), the authors would like to update the Acknowledgements section as follows:

**Acknowledgements**

The authors would like to thank Dr. Kevin J. Tracey, Dr. Ravi Sitapara and Ms. LeeAnne Daley for the insightful discussion, Dr. Sarah Oelsner for critical reading of the manuscript, and Dr. Douglas David Thomas for contributing to the concept and the protocol on the involvement of NO in this project and for providing numerous feedback on the presentation of this manuscript. A portion of the publication fee was defrayed by Chimerix Inc.

The original article has been corrected.
