# Supplementary material for: 2-O, 3-O desulfated heparin (ODSH) increases bacterial clearance and attenuates lung injury in cystic fibrosis by restoring HMGB1-compromised macrophage function
Source: Mol Med. 2021 Jul 16;27:79. doi: 10.1186/s10020-021-00334-y (PMC8283750; doi:10.1186/s10020-021-00334-y)
Supplement: Supplementary file 1 — Additional file 1: Figure 1. Western blot immunoreactive images of airway HMGB1 in PA infected mice in the absence or presence of ODSH. Figure 2. ODSH does not significantly improve intrinsic phagocytic ability of macrophages in CF mice. [file 10020_2021_334_MOESM1_ESM.docx]

**Additional file 1**

**Figure 1. Western blot immunoreactive images of airway HMGB1 in PA-infected mice in the absence or presence of ODSH.**

C57BL/6 mice (WT) and CFTR-/- mice (CF) were randomized to receive i.p. of saline or ODSH at 8.3 or 25 mg/kg every 12 hours. After 24 hours, mice were intratracheally inoculated with *Pseudomonas aeruginosa* (PA). BALF was collected 18 hours after infection and was analyzed for the levels of airway HMGB1 by Western blot analysis.

**
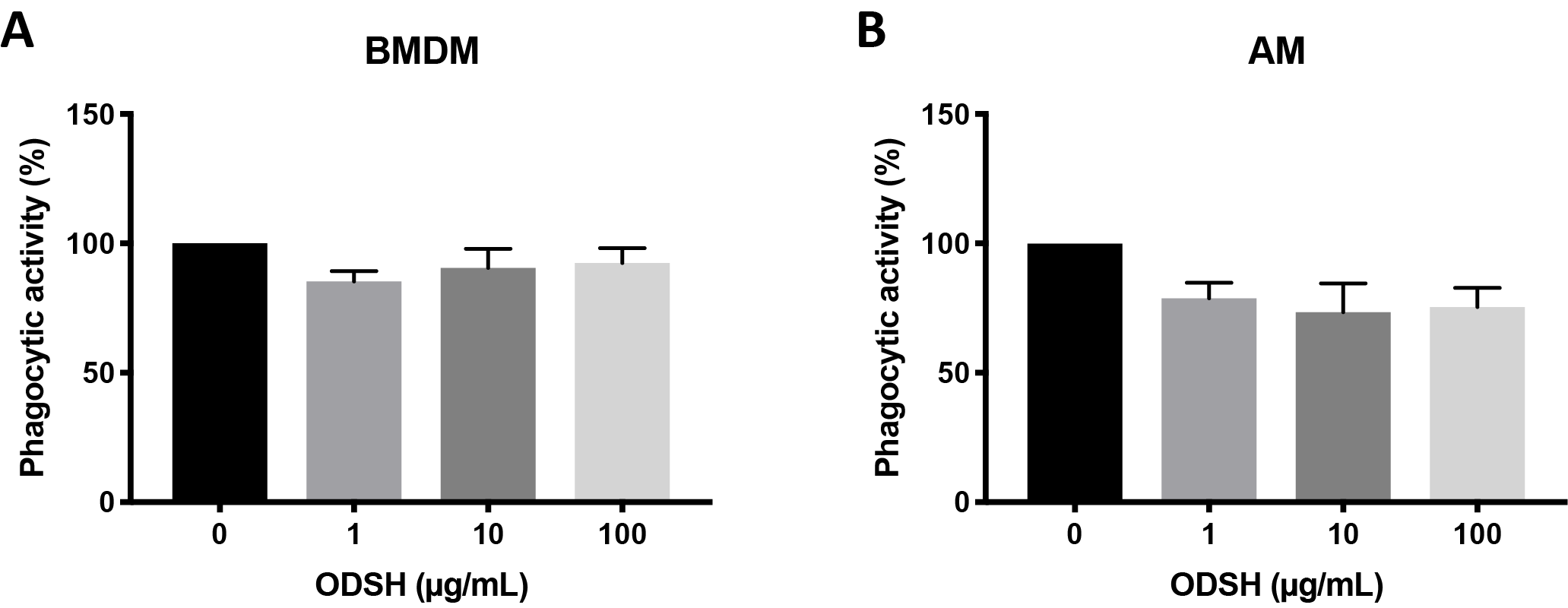
**

**Figure 2. ODSH does not significantly improve intrinsic phagocytic ability of macrophages in CF mice.** BMDM (A) and AM (B) isolated from CFTR^-/-^ mice (CF, n=3) were cultured, then treated without or with ODSH at 1, 10, 100 µg/mL for 24 hours. Phagocytosis assay was performed on the macrophages by incubating with FITC-labeled latex mini-beads for 1 hour and stained with DAPI and phalloidin to visualize the cells. Phagocytic ability of the cells was represented as the percentage of mini-beads that were phagocytosed by the cells normalized to the control group. Data were represented by mean ± SEM of at least three independent experiments.
